# Perils of inappropriate antibiotic exposure in cancer patients. A call to fast-acting synergy

**DOI:** 10.1186/1753-6561-5-S6-P52

**Published:** 2011-06-29

**Authors:** L Pagani

**Affiliations:** 1 Bolzano Central Hospital, Unit for Hospital Antimicrobial Chemotherapy, Bolzano, Italy

## Introduction / objectives

Use and overuse of antibiotics are among the main driving factors for the emergence of antimicrobial resistance (AMR). However, oncology patients often require prolonged administration of broad spectrum antimicrobials.

## Methods

A literature search was performed through PubMed from 1 January 2006 to 31 December 2010 to identify review papers, international guidelines, and standardised protocols for infection prophylaxis and therapy in oncology patients. Keywords used were: "cancer", "infection", "febrile neutropenia", and “antimicrobial resistance”.

## Results

The search yielded 309 papers; 60 were selected according to the above-mentioned criteria. The main gaps identified in all retrieved papers were: limited relevance of both updated local patterns of AMR and antimicrobial pharmacokinetics and pharmacodynamics; and no integration with infection control policies to prevent transmission of multidrug-resistant organisms (MDRO). Monitoring and feedback of AMR data are not only the hallmark of infection control — they are also necessary to tailor subsequent empirical regimens; pharmacokinetics and pharmacodynamics can optimize drug choice and daily dosing, according to the target site of infection. Although antimicrobial stewardship programmes (ASP) are recognized as effective tools to optimize antimicrobial usage, tackle AMR, and improve clinical outcomes on an institutional basis, none has been implemented in oncology settings as yet, and the concept itself of ASP is lacking for this patient population.

## Conclusion

Oncology patients would benefit from a multidisciplinary approach integrating local AMR patterns, pharmacokinetics and pharmacodynamics, strict infection control policies, and reliable diagnosis through novel technologies. At last, they should not be excluded from the assets that targeted interventions provided by ASP can offer.

## Disclosure of interest

None declared.

